# Ultrasound imaging in an experimental model of fatty liver disease and cirrhosis in rats

**DOI:** 10.1186/1746-6148-6-6

**Published:** 2010-01-29

**Authors:** Andréia S Lessa, Bruno D Paredes, Juliana V Dias, Adriana B Carvalho, Luiz Fernando Quintanilha, Christina M Takiya, Bernardo R Tura, Guilherme FM Rezende, Antonio C Campos de Carvalho, Célia MC Resende, Regina CS Goldenberg

**Affiliations:** 1 Department of Internal Medicine, School of Medicine, Federal University of Rio de Janeiro, Clementino Fraga Filho University Hospital, Rua Prof. Rodolpho Paulo Rocco, 255, Rio de Janeiro, 21941-913, Brasil; 2 Carlos Chagas Filho Biophysics Institute, Federal University of Rio de Janeiro, Av. Carlos Chagas Filho, 373, Bloco G, Sala G2-053, Rio de Janeiro, RJ, 21941-902, Brasil; 3 Department of Radiology, School of Medicine, Federal University of Rio de Janeiro, Clementino Fraga Filho University Hospital, Rua Professor Rodolpho Paulo Rocco, 255, Rio de Janeiro, 21941-913, Brasil; 4 Department of Histology and Embryology, Institute of Biomedical Sciences, Federal University of Rio de Janeiro, Av. Carlos Chagas Filho, 373, Bloco F2-024, Rio de Janeiro, RJ, 21941-902, Brasil; 5 National Institute of Cardiology, Rua das Laranjeiras, 374, 2° andar, Rio de Janeiro, RJ, 22240-006, Brasil

## Abstract

**Background:**

Domestic dogs and cats are very well known to develop chronic hepatic diseases, including hepatic lipidosis and cirrhosis. Ultrasonographic examination is extensively used to detect them. However, there are still few reports on the use of the ultrasound B-mode scan in correlation with histological findings to evaluate diffuse hepatic changes in rodents, which represent the most important animal group used in experimental models of liver diseases. The purpose of this study was to determine the reliability of ultrasound findings in the assessment of fatty liver disease and cirrhosis when compared to histological results in Wistar rats by following up a murine model of chronic hepatic disease.

**Results:**

Forty Wistar rats (30 treated, 10 controls) were included. Liver injury was induced by dual exposure to CCl_4 _and ethanol for 4, 8 and 15 weeks. Liver echogenicity, its correlation to the right renal cortex echogenicity, measurement of portal vein diameter (PVD) and the presence of ascites were evaluated and compared to histological findings of hepatic steatosis and cirrhosis. Liver echogenicity correlated to hepatic steatosis when it was greater or equal to the right renal cortex echogenicity, with a sensitivity of 90%, specificity of 100%, positive and negative predictive values of 100% and 76.9% respectively, and accuracy of 92.5%. Findings of heterogeneous liver echogenicity and irregular surface correlated to liver cirrhosis with a sensitivity of 70.6%, specificity of 100%, positive and negative predictive values of 100% and 82.1% respectively, and accuracy of 87.5%. PVD was significantly increased in both steatotic and cirrhotic rats; however, the later had greater diameters. PVD cut-off point separating steatosis from cirrhosis was 2.1 mm (sensitivity of 100% and specificity of 90.5%). One third of cirrhotic rats presented with ascites.

**Conclusion:**

The use of ultrasound imaging in the follow-up of murine diffuse liver disease models is feasible and efficient, especially when the studied parameters are used in combination. The potential implication of this study is to provide a non-invasive method that allows follow-up studies of fatty liver disease and cirrhosis of individual rats for pre-clinical drug or cell based therapies.

## Background

Domestic dogs and cats are very well known to develop chronic hepatic diseases. Hepatic lipidosis (HL) or fatty liver is an important liver disease in cats. It is characterized by excess fat accumulation in the liver and seems to have no age, breed, or gender predilection [1,2]. HL is more common in obese or previously obese cats which have undergone brief periods of anorexia or food deprivation [3]. Obesity in cats has been described as the most prevalent nutritional disease of the domestic cat in the United States of America [4]. Cornelius and DeNovo [5] reported HL as the underlying disease process in 12.5% of 80 cats with icterus.

Chronic hepatitis is a well-recognized problem in canine practice and affects dogs of many breeds [6]. However, knowledge of its etiology in dogs remains limited [7]. If chronic hepatitis is not treated, it can lead to the development of liver cirrhosis, which is characterized by architectural distortion of lobules, bile duct hyperplasia, nodular regeneration and fibrosis [7].

Murine models of diffuse liver disease can be used as a base for pre-clinical studies [8]. Radiological methods such as ultrasound (US) brightness-mode (B-mode) imaging [1,2,9-11], duplex Doppler US [12], computed tomography perfusion [13] and magnetic resonance imaging (MRI) [14,15] have been used to detect and to follow-up experimental diffuse liver disease models. Additionally, Quintanilha *et al *[16] utilized US B-mode imaging in a longitudinal study to follow-up the effects of bone marrow stem cell therapy in rats submitted to liver injury, while Ju *et al *[17] prospectively tracked stem cells labeled with super paramagnetic particles transplanted intrasplenically in cirrhotic rats by MRI.

US examination has been extensively used to detect fatty liver disease and cirrhosis [18-20]. In cats and dogs, fatty liver disease can be diagnosed by the observation of an increased hepatic echogenicity, characterized by hyperechoic liver parenchymal texture compared to the renal cortex, and decreased visualization of deeper structures, such as the diaphragm and small peripheral vessels [21,22]. Cirrhosis, on the other hand, can be detected by a coarse and heterogeneous parenchymal echogenicity, irregular or nodular liver surface [21-23], focal regenerative nodules [21,24], decreased right lobe to caudate lobe ratio, indicating volume redistribution [25], and by indirect evidence of portal hypertension, such as increased portal vein diameter (PVD), presence of collateral vessels, splenomegaly and ascites [21,23,26].

Even though there are a number of studies that use US B-mode to evaluate liver diseases in rodents [9,27,28], few of these studies have statistically correlated US findings to the histological alterations present in liver parenchyma [29-31]. Lee *et al *[29] compared histopathological findings to the US B-mode quantitative parameter Mean Grey Level in CCl_4_-induced liver cirrhosis, while Matsuhashi *et al *[30] and Layer *et al *[31] correlated histology to Hepatic Ultrasound Speed and Texture Analysis respectively.

However, regardless of the fact that there is a correlation between quantitative B-mode methods and the histological features of cirrhosis, qualitative ultrasonography, which is the most common evaluation method of liver diseases in clinical practice both in animals and in humans, remains to be validated as a technique for the non-invasive diagnosis of such diseases in rodents. The purpose of our study was to determine the reliability of US qualitative findings in the assessment of fatty liver disease and cirrhosis in Wistar rats in comparison to histological results by following up a murine model of hepatic disease induced by dual exposure to CCl_4 _and ethanol.

## Methods

### Animals

This investigation was performed in agreement with the Guide for Care and Use of Laboratory Animals [DHHS Publication No. (NIH) 85-23, revised 1996, Office of Science and Health Reports, Bethesda, MD 20892] and the authors have received approval from the Institutional Animal Care and Use Committee (protocol #021).

Female Wistar rats with 6 - 12 months of age, weighing between 160 - 180 g were housed at controlled temperature (23°C) with daily exposure to a 12:12 light-dark cycle.

### Model of Experimental Cirrhosis

Animals (n = 40) were randomly divided into two groups: the Control group (n = 10) was fed with standard rat chow and water, and the Experimental group (n = 30) had chronic liver damage induced according to the protocol previously described by Dias et al [10], Quintanilha et al [16] and Carvalho et al [32]. Briefly, liver damage was induced with injections of a 20% solution of carbon tetrachloride (CCl_4_) (1:5 in olive oil, dose of 0.05 mL/kg) intraperitoneally three times a week on alternate days combined with an alcoholic liquid diet in accordance with the AIN-93 guidelines [33] over 15 weeks.

### Ultrasound Analysis

The Control and the Experimental groups were examined at the beginning of the experiment and after 4, 8 and 15 weeks of liver injury induction. Animals were anesthetized using ketamine (0.5 mL/kg) and xilazin (1 mL/kg) and had the abdomen shaved to reduce imaging artifacts in the ultrasonographic examination. A sound-conducting gel (Carbogel^®^, Brazil) was applied and an US examination was performed by two blinded expertise authors by consensus (reader 1, A.S.L., with 7 years experience, reader 10, C.M.C.R., with 26 years experience) by using a multifrequency linear transducer (7.5 to 10 MHz) and a Caris Plus^® ^ultrasound equipment (Esaote, Italy). All imaging was performed in fundamental brightness mode (B-mode). Two-dimensional B-mode image plans were acquired with optimization of the gain and the time gain compensation settings, which were kept constant throughout the experiment. The acoustic focus was placed in the center of the target organ (liver) and in the largest transverse cross section of the spleen. The animals were examined in supine position to assess the liver and the portal vein, and in the right posterior oblique position to assess the spleen. These organs were evaluated by multiple transversal and longitudinal scans.

Ultrasonographic findings were analyzed based on our previous observations and on criteria for US diagnosis in humans according to the following classification:

A. Changes in the liver echogenicity, classified into four patterns: (1) homogeneous liver parenchyma with medium level echogenicity and a regular hepatic surface; (2) diffusely increased parenchymal echogenicity, reduced visualization of the diaphragm and small peripheral vessels in the liver with no change on liver surface; (3) discrete coarse and heterogeneous parenchymal echogenicity, dotted or slightly irregular liver surface; (4) extensive coarse and heterogeneous parenchymal echogenicity, irregular or nodular hepatic surface reflecting the presence of underlying regenerative nodules;

B. Inversion of the echographic relationship between the liver and the renal cortex. This finding was considered to be positive if the echogenicity of the liver parenchyma was greater or equal to that of the right renal cortex;

C. Increased PVD. It was measured in the mid-point of the main portal vein by using calipers of the scanner. PVD was considered to be abnormal if equal or greater than 2.1 mm;

D. Presence of ascites;

E. Spleen area. It was directly calculated by the US software after organ surface was delineated by the examiner.

### Histological analysis

Rats from the Experimental and Control Groups were sacrificed on the 4^th ^(n = 6), 8^th ^(n = 7) and 15^th ^(n = 17) week of the cirrhosis induction protocol. The liver was removed from the abdominal cavity for macroscopic post mortem analysis. For microscopic examination, liver tissue was fixed for 5 h in Gendre's solution followed by overnight incubation in 10% buffered formalin solution (pH 7.2) and embedded in paraffin. Liver samples were sectioned (5 μm) and stained with hematoxilin and eosin (H&E) [34] or Sirius Red [35] according to standard protocols.

The histological diagnosis of fatty liver disease and cirrhosis was defined by a single blinded experienced pathologist based on the hepatic steatosis grading established by Ishak *et al *[36] and on the scoring system for hepatic fibrosis described by Plummer *et al *[37], respectively. Histology was used as the gold standard for both the diagnosis of fatty liver disease and cirrhosis and for the comparison with US results.

### Statistical Analysis

The sensitivity, specificity, positive predictive value, negative predictive value and accuracy of the US findings for the detection of fatty liver disease and cirrhosis were calculated in comparison to the histological diagnosis [38]. PVD values were analyzed using analysis of variance (ANOVA) with Tukey's post-test for multiple comparisons. The value of *p *< 0.01 was considered statistically significant. Data are presented as mean ± SD. We also performed a Receiver Operating Characteristic (ROC) analysis of these data.

## Results

### Ultrasound Imaging

All animals were examined by US imaging in the beginning of experiment and after 4, 8 and 15 weeks of liver injury induction and the alterations found in the Experimental group were homogeneous in each examination time point. These alterations were represented by changes in liver echogenicity, in the relationship between liver parenchyma and renal cortex echogenicity, increased values of PVD and higher incidence of ascites when compared to the Control group (Table 1). A subset of animals in the Experimental group was sacrificed after 4 (n = 6) and 8 (n = 7) weeks of liver injury induction for histological studies, while the remaining animals (n = 17) were sacrificed after 15 weeks.

**Table 1:** Ultrasound findings over the time of experiment observed in Experimental group compared to Control group

US findings/Time of experiment	Control Group at any time (n = 10)	Experimental Group		
		**4 weeks (n = 6)**	**8 weeks (n = 7)**	**15 weeks (n = 17)**

Homogeneous liver parenchyma of medium level echogenicity (pattern 1)	10	3	0	0

Diffusely increased parenchymal echogenicity (pattern 2)	0	3	3	0

Discrete coarsened and heterogeneous parenchyma (pattern 3)	0	0	4	5

Extensive coarsened and heterogeneous parenchyma (pattern 4)	0	0	0	12

L-Echo<R-Echo	10	3	0	0

L-Echo=R-Echo	0	2	4	8

L-Echo>R-Echo	0	1	3	9

Liver isoechoic nodule	0	0	0	2

Liver hypoechoic nodule	0	0	0	1

PVD< 2.1 mm	10	6	6	0

PVD ≥ 2.1 mm	0	0	1	17

Mild or transient ascites	0	0	0	2

Moderate or marked ascites	0	0	0	3

PVD: Portal Vein Diameter; L-Echo: Liver echogenicity; R-Echo: Renal cortex echogenicity.

### Histological Analysis

**Figure 1:**
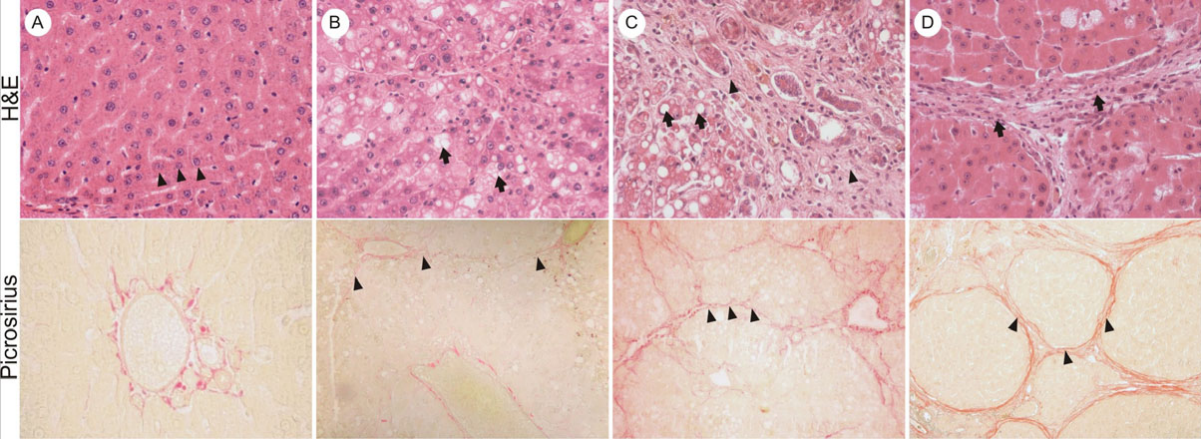
**Histological findings observed over the experiment**. (a) Microscopy of Control group (normal liver): Hematoxilin/Eosin (H&E) section (magnification 100×) shows hepatocytes radially arranged in plates aligned to sinusoids (arrowheads). Sirius red (magnification 100×) staining shows only vessels stained in red (portal triad - PT), which is a normal place of collagen deposit. (b) After 4 weeks of experiment: H&E section (magnification 100×) shows moderate steatosis (arrows). Sirius red (magnification 100×) staining shows focal perivenular and pericellular fibrosis (arrowheads). (c) After 8 weeks of experiment H&E section (magnification 100×) shows moderate steatosis (arrows) with some fibrosis (arrowheads) but preserved liver parenchymal architecture. Sirius red (magnification 100×) staining some fibrous septa linking centrolobular veins with portal tracts (arrowheads). (d) After 15 weeks of protocol: H&E section (magnification 100×) shows macronodular cirrhosis, characterized by thick collagen septa, forming regenerative nodules, global architectural distortion, some inflammatory infiltrate (arrow) and steatosis. Sirius red (magnification 100×) staining shows thick collagen septa (arrowheads) linking portal tracts, centrolobular veins, and portal tracts to centrolobular veins, forming regenerative nodules (RN).

Control group showed a normal architecture with hepatocytes radially arranged in plates aligned to sinusoids converging to centrolobular veins and without evidence of fibrosis (Figure 1a). On the other hand, the Experimental group showed liver parenchymal alterations that correlated to the time of injury induction. After 4 weeks (n = 6), half of the animals presented with mild steatosis without fibrous septa and the other half showed mild to moderate steatosis with minimal focal pericellular and perivenular fibrosis (Figure 1b). After 8 weeks (n = 7), all the animals showed moderate to marked steatosis with some fibrous septa linking centrolobular veins to portal tracts, although liver parenchymal architecture was preserved (Figure 1c). After 15 weeks of induction (n = 17), all rats had macronodular cirrhosis, characterized by global architectural distortion and formation of regenerative nodules surrounded by collagen septa and containing some inflammatory infiltrate (Figure 1d). Mild to moderate steatosis was also present.

The prevalence of fatty liver disease among the 30 rats in the Experimental group was 100%, while the prevalence of cirrhosis was 56.7% (17 out of 30) when considering all the time points examined together. The lower prevalence of cirrhosis is expected since animals take longer periods of time to fully develop the histological alterations found in this disease. Accordingly, if we consider only the subset of animals that were injured for 15 weeks, the prevalence of cirrhosis was also 100%. No animals in the Control group presented with fatty liver disease or cirrhosis.

### Correlation between Ultrasound Findings and Histological Diagnosis

**Figure 2:**
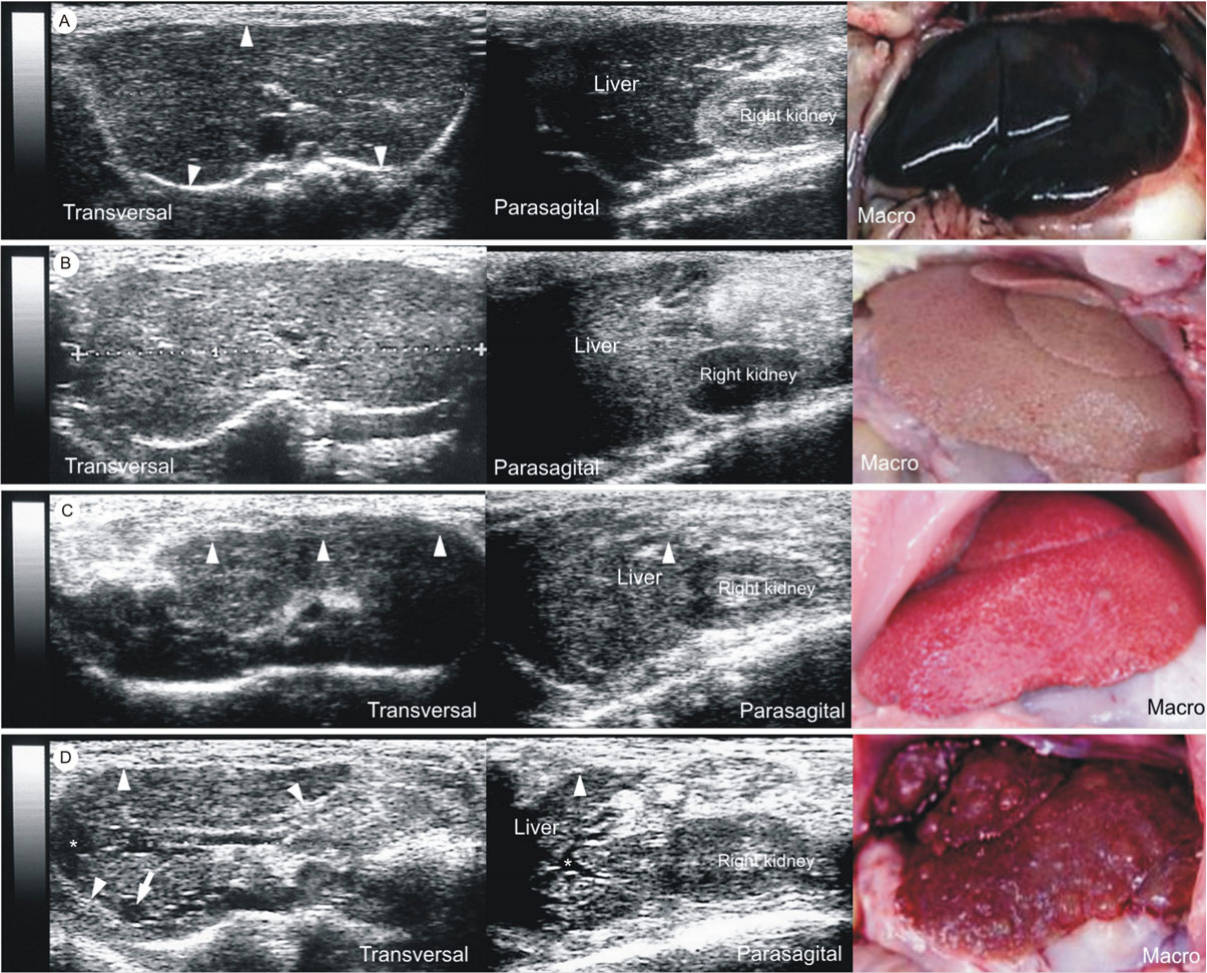
**Correlation between ultrasound and histological findings**. (a) Sonograms and macroscopy of Control group (normal liver): Transversal sonogram demonstrates homogeneous liver parenchyma, with medium level echogenicity and a regular hepatic surface (arrowheads). Parasagittal sonogram presents right renal cortex more echogenic than liver. Macroscopy shows smooth surface, brownish-red color and friable consistency. (b) Sonograms and macroscopy of fatty liver: Transversal sonogram presents diffusely increased parenchymal echogenicity. Parasagittal sonogram demonstrates hepatic echogenicity greater than that of right renal cortex. Macroscopy exhibits wrinkled surface, yellowish color and friable consistency. (c) Sonograms and macroscopy of liver carrying steatosis and fibrosis: Transversal sonogram shows discrete coarse and heterogeneous parenchymal echogenicity and the liver surface as a dotted or slightly irregular line (arrowheads). Parasagittal sonogram presents hepatic echogenicity equal to the right renal cortex echogenicity and a slightly irregular liver surface (arrowhead). Macroscopy shows a slightly irregular surface, pale red color and more rigid consistency. (d) Sonograms and macroscopy of cirrhotic liver: Transversal sonogram presents extensive coarse and heterogeneous parenchymal echogenicity, extremely irregular hepatic surface (arrowheads), a hypoechoic regenerative nodule (arrow) and mild ascites (*). Parasagittal sonogram shows hepatic echogenicity slightly greater than that of the right renal cortex, extremely irregular liver surface (arrowhead) and mild ascites (*). Macroscopy reveals extremely irregular surface, reflecting the presence of underling regenerative nodules, reddish color and rigid consistency.

Ultrasound and histological findings among the experimental groups were compared in the different time points (Table 2, Figure 2). Samples of acquired US data used to perform correlation analysis can be seen in additional files (additional file 1, 2, 3, 4 and 5)

**Table 2:** Ultrasound findings compared to histological diagnosis

Histologic Diagnosis				
**US findings**	**Normal (n = 10)**	**Mild steatosis without fibrous septa (n = 3)**	**Mild to marked hepatic steatosis with various degrees of fibrosis but intact liver parenchymal architecture (n = 10)**	**Macronodular cirrhosis with hepatic steatosis(n = 17)**

Pattern 1 L-Echo L-Echo < R-Echo	10	3	0	0

Pattern 1 L-Echo L-Echo ≥ R-Echo	0	0	0	0

Pattern 2, 3 or 4 L-Echo L-Echo < R-Echo	0	0	0	0

Pattern 2 L-Echo L-Echo ≥ R-Echo	0	0	6	0

Pattern 3 L-Echo L-Echo ≥ R-Echo	0	0	4	5

Pattern 4 L-Echo L-Echo ≥ R-Echo	0	0	0	12

Liver nodules	0	0	0	3 (regenerative nodules)

PVD < 2.1 mm	10	3	9	0

PVD ≥ 2.1 mm	0	0	1	17

Presence of ascites	0	0	0	5

L-Echo: Liver echogenicity; Pattern 1: normal echogenicity, pattern 2: diffusely increased echogenicity, pattern 3: slightly coarse echogenicity, and pattern 4: coarse and heterogeneous echogenicity; R-Echo: Renal cortex echogenicity; PVD: Portal Vein Diameter.

#### Liver echogenicity

The increase in liver echogenicity compared to right renal cortex echogenicity (L-Echo ≥ R-Echo) had a sensitivity of 90% (27 out of 30), specificity of 100% (10 out of 10), positive predictive value of 100% (27 out of 27), negative predictive value of 76.9% (10 out of 13) and accuracy of 92.5% (37 out of 40) for the detection of fatty liver disease and were well correlated with patterns 2 and 3 of liver echogenicity. The 3 rats which appeared to be normal in ultrasound imaging showed mild steatosis in microscopy. Control animals were all classified as pattern 1 and presented with normal liver parenchyma.

Moreover, pattern 4, characterized by extensive coarse and heterogeneous parenchymal echogenicity and irregular or nodular hepatic surface, was always associated with alteration of the echographic relationship between the liver and the right renal cortex. It had a sensitivity of 70.6% (12 out of 17), specificity of 100% (23 out of 23), positive predictive value of 100% (12 out of 12), negative predictive value of 82.1% (23 out of 28) and accuracy of 87.5% (35 out of 40) for the detection of liver cirrhosis.

#### Portal Vein Diameter (PVD)

**Figure 3:**
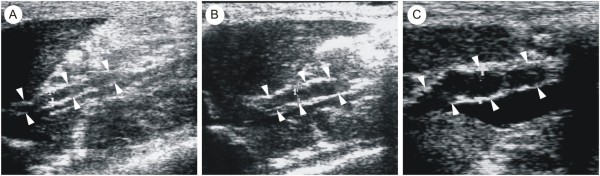
**Portal vein widening over the experiment**. (a) Parasagittal sonogram shows a straight and thin portal vein (diameter = 1.5 mm) in a Control group rat (arrowheads). (b) Parasagittal sonogram demonstrates a slightly tortuous and thin portal vein (diameter = 2.0 mm) in a rat with hepatic steatosis and fibrosis (arrowheads). (c) Parasagittal sonogram presents a tortuous and wide portal vein (diameter = 2.4 mm) in a cirrhotic rat (arrowheads).

**Figure 4:**
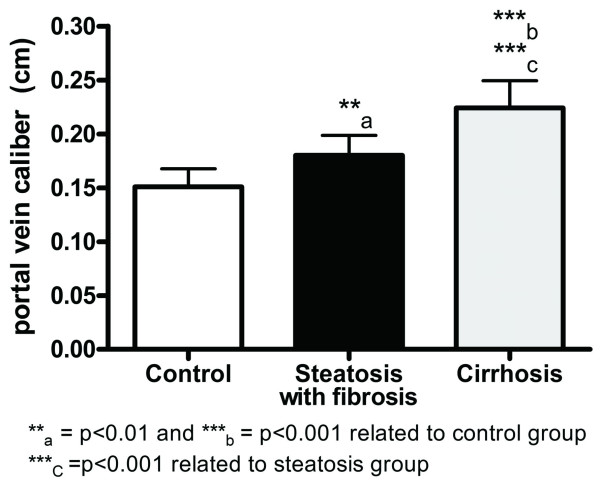
**Comparison between portal vein diameter and histological findings**. Quantification of portal vein diameter shows increase in this value in the group of cirrhotic rats (mean = 2.2 mm) and in the group of rodents with steatosis and fibrosis (mean = 1.8 mm) compared to the Control group (mean = 1.5 mm). Data are shown as mean ± SD and the test utilized was ANOVA with the Tukey's post-test for multiple comparisons.

In the Experimental group, portal vein became wider and tortuous during hepatic disease development when compared to Control group (Figure 3). We analyzed PVD values among rats with cirrhosis, rats with steatosis and fibrosis and rats from the Control group. The results found were 2.2 ± 0.2 mm, 1.8 ± 0.2 mm and 1.5 ± 0.2 mm, respectively. PVD was significantly higher in cirrhotic rats and in rats with steatosis and fibrosis in comparison to the Control group (Figure 4), suggesting the presence of portal hypertension.

**Figure 5:**
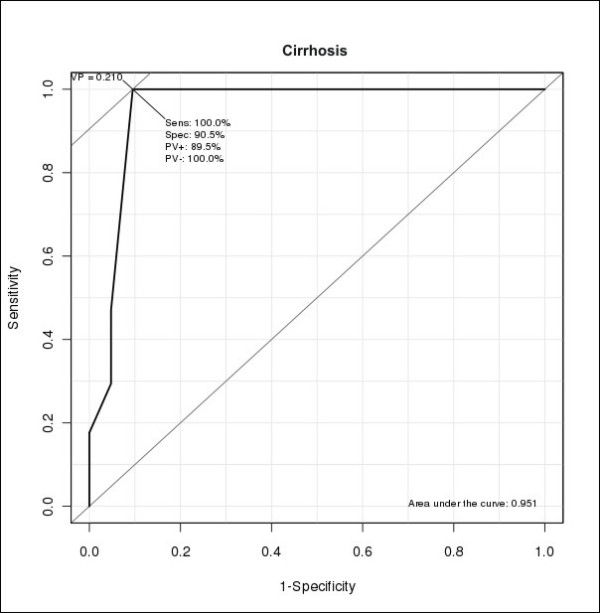
**ROC (Receiver Operating Characteristic) analysis of Portal Vein Diameter (PVD) values**.

After Receiver Operating Characteristic (ROC) analysis of these data (Figure 5), we found that PVD values equal or greater than 2.1 mm had sensitivity of 100%, specificity of 90.5%, positive predictive value of 89.5% and negative predictive value of 100% for the detection of cirrhosis. Thus, this value can be considered a cut-off point for the diagnosis of cirrhosis in rats.

#### Presence of ascites and spleen measurements

Furthermore, the occurrence of ascites could be easily identified, even when small quantities of fluid were present in the abdominal cavity. This alteration was observed in 5 out of 17 cirrhotic rats (34%).

We also compared the spleen area among rats with cirrhosis, rats with steatosis and fibrosis and rats from the Control group. The results found were 1.19 ± 0.16 cm^2^, 1.16 ± 0.14 cm^2 ^and 1.18 ± 0.16 cm^2^, respectively. There were no statistical differences among the three groups analyzed, indicating that splenomegaly was not a prominent feature in cirrhotic rats.

## Discussion

In this work we demonstrated that qualitative ultrasound analysis is sensitive and specific enough to diagnose fatty liver disease and cirrhosis in rats and, therefore, can reliably substitute histological diagnosis in experimental liver disease models. Ultrasound imaging has many advantages, namely its low cost, extensive availability and the fact that it can be easily done in murine models. Only slight sedation and abdominal shaving are required, not demanding total immobility or apnea while obtaining the images [39], therefore, consisting in a low risk procedure. Moreover, it is an accurate, innocuous and non-invasive technique and it is the most commonly used method for the investigation of liver diseases in humans and small animals [1,18-21,40,41].

Although ultrasound is an essentially subjective diagnostic method, its validity as an imaging technique for liver evaluation has been extensively described in the literature for humans [40,42], dogs [21-23] and cats [1,21]. In our study, we focused our attention in a rodent model of liver disease, since these are the most commonly used models in pre-clinical studies [37,43-46]. The rat model was chosen because they are larger than mice, facilitating US imaging procedure.

Healthy rats showed homogeneous liver parenchyma, with medium level echogenicity and regular liver surface, as previously described in humans [47], dogs [21,23,48] and cats [2,21]. Liver echogenicity was lower in comparison to the right renal cortex, a finding that contrasts with humans [47], but is in accordance to what has been described in dogs [49]. In cats, renal cortical echogenicity varies in normal animals [50]. Fatty liver disease in rats was characterized by homogeneous and diffusely increased echogenicity ("bright liver"), equal or greater to the right renal cortex, reduced visualization of the diaphragm and of small peripheral vessels, and by no changes in liver surface, as described in humans [51], dogs [21,23], and cats [1,21].

Liver echogenicity increases due to the presence of fatty infiltration and/or fibrosis [21,23], changing the relation between liver and right renal cortex echogenicity [21,22]. This finding had high sensitivity (90%), specificity (100%), positive and negative predictive values (100% and 76.9%), and accuracy (92.5%) for the detection of fatty liver disease, values that were compatible with the ones previously described in the literature. The increased liver echogenicity has been reported to have sensitivity of 60 to 82% [1,23,52], specificity of 97 to 100% [1,52] and positive predictive value of 96 to 100% [1,52] for detection of fatty liver disease. However, it is important to exclude kidney diseases when utilizing this approach [23].

Three rats with mild hepatic steatosis appeared to be normal in US examination, which is also comparable to previous reports in the literature [23,52]. US has been reported to have sensitivity of 90 - 91% for the detection of moderate to marked steatosis, but its sensitivity decreases to 30 - 64% when steatosis is mild [23,52].

The finding of an extensive coarse and heterogeneous parenchyma associated to irregular or nodular hepatic surface proved to be relevant in the detection of cirrhosis with sensitivity of 70.6%, specificity of 100%, positive predictive value of 100%, negative predictive value of 82.1% and accuracy of 87.5%, as seen in humans [40,53] and in dogs [23]. Its presence implied a definitive diagnosis of cirrhosis since there were no false negative results. Therefore, US imaging can consistently substitute histology to diagnose cirrhosis in rats, constituting a useful method to avoid unnecessary animal sacrifice or repeated liver biopsies when following long experimental protocols.

The evaluation of portal hypertension is also important to assess the severity of liver diseases [20,21,40] and one of its most important signs is the widening of the portal vein [20,21,47]. PVD was statistically different when comparing cirrhotic to non-cirrhotic rats (p < 0.001). An increase in its caliber when equal or superior to 2.1 mm can be considered a relevant ultrasound finding with sensitivity of 100%, specificity of 90.5%, positive predictive value of 89.5% and negative predictive value of 100% for the detection of cirrhosis. There was one false positive, a rat bearing liver steatosis with fibrosis. There were no false negatives since none of the rats with PVD lower than 2.1 mm were cirrhotic. Additionally, PVD measurements were unable to distinguish normal from steatotic livers because of the considerable overlap between PVD values encountered in normal rats compared to rats carrying steatosis and fibrosis. This fact is, nonetheless, expected since fatty liver disease does not lead to portal hypertension and, consequently, to the widening of the portal vein. Doppler evaluation of the portal vein was also tried, yet it was not possible due to technical difficulties. The caliper could not be stably positioned inside such a small vessel, we could not induce apnea and whirl flow was found even in normal rats, causing an oscillation between hepatopetal and hepatofugal flow.

The presence of ascites, which is also an important indicator of the severity of liver disease, could be detected even when the amount of fluid present in the peritoneal cavity was small, as previously reported by Vörös et al [54]. Among the imaging diagnostic methods, ultrasound is considered ideal for the study of ascites [6,21,23]. In our study, its presence invariably corresponded to cirrhosis in histology, although only 5 animals presented with ascites out of 17 cirrhotic rats. This was, however, expected since ascites is only observed when cirrhosis is decompensated. In addition, although splenomegaly should be expected in severe cirrhosis with portal hypertension, there were no statistical differences in splenic area among the three groups analyzed, indicating that splenomegaly was not a prominent feature in our study of cirrhotic rats.

## Conclusion

The use of ultrasound imaging in the diagnosis of murine liver disease models is feasible and efficient, especially when different parameters (liver echogenicity and its correlation to that of the right renal cortex, PVD and the presence of ascites) are used in combination. The implication of this study is to certify that fatty liver disease and cirrhosis can be reliably diagnosed by a non-invasive method that allows adequate long-term follow-up, which is usually needed in pre-clinical drug or cell-based therapy studies.

## Competing interests

The authors declare that they have no competing interests.

## Authors' contributions

BDP, LFQM, ABC, GFMR, ACCC and RCSG designed the study, performed the pilot studies and helped to draft the manuscript. ASL and CMCR carried out the ultrasonography and drafted the manuscript. BPD, JVD, CMT and BRT carried out analysis and interpretation of the data and helped to draft the manuscript. All authors revised and approved the final manuscript.

## Supplementary Material

Additional file 1 **Ultrasound imaging of Control group (transversal and parasagittal views)**. The exam begins making transversal scanning, which shows homogeneous liver parenchyma, with medium level echogenicity and a regular hepatic surface. In the sequence, the parasagittal scanning shows right renal cortex more echogenic than liver. Author: Lessa AS. Length: 31 seconds. Size: 2.65 MB. Click here for file

Additional file 2 **Ultrasound imaging of steatotic liver (transversal view)**. Transversal scanning shows diffusely increased parenchymal echogenicity. Author: Lessa AS. Length: 35 seconds. Size: 3.02 MB. Click here for file

Additional file 3 **Ultrasound imaging of steatotic liver (parasagittal view)**. Parasagittal sacanning shows hepatic echogenicity slightly greater than that of right renal cortex. Author: Lessa AS. Length: 10 seconds. Size: 0.39 MB. Click here for file

Additional file 4 **Ultrasound imaging of cirrhotic liver (transversal view)**. Transversal scanning shows marked ascites, extensive coarse and heterogeneous parenchymal echogenicity and extremely irregular hepatic surface. Author: Resende CMC. Length: 35 seconds. Size: 3.06 MB. Click here for file

Additional file 5 **Ultrasound imaging of cirrhotic liver (parasagittal view)**. Parasagittal scanning shows hepatic echogenicity equal to that of the right renal cortex, extremely irregular liver surface, tortuous and wide portal vein and marked ascites. Author: Resende CMC. Length: 70 seconds. Size: 6.06 MB. Click here for file

## References

[B1] Yeager AE Mohammed H Accuracy of ultrasonography in the detection of severe hepatic lipidosis in cats Am J Vet Res 1992 53 597 599 1586035

[B2] Nicoll RG O'Brien RT Jackson MW Qualitative ultrasonography of the liver in obese cats Vet Radiol Ultrasound 1998 39 47 50 10.1111/j.1740-8261.1998.tb00324.x 9491517

[B3] Center AS Crawford MA Guida L Erb HN King J A retrospective study of 77 cats with severe hepatic lipidosis: 1975-1990 J Vet Intern Med 1993 7 349 359 8114031 10.1111/j.1939-1676.1993.tb01030.x

[B4] Scarlett JM Donoghue S Saidla J Wills J Overweight cats: prevalence and risk factors Int J Obes Relat Metab Disord 1994 18 Suppl 1 22 28 8087161

[B5] Cornelius LM DeNovo RC KirK RW Icterus in Cat Current veterinary therapy 1983 8 Philadelphia: WB Saunders Co 822 827

[B6] Raffan E McCallum A Scase TJ Watson PJ Ascites is a negative prognostic indicator in chronic hepatitis in dogs J Vet Intern Med 2009 23 63 66 10.1111/j.1939-1676.2008.0230.x 19175722

[B7] Sevelius E Diagnosis and prognosis of chronic hepatitis and cirrhosis in dogs J Small Anim Pract 1995 36 521 528 10.1111/j.1748-5827.1995.tb02801.x 8926720

[B8] Bujanda L Hijona E Larzabal M Beraza M Aldazabal P Garcia-Urkia N Sarasqueta C Cosme A Irastorza B Gonzalez A Arenas JI Jr Resveratrol inhibits nonalcoholic fatty liver disease in rats BMC Gastroenterol 2008 8 40 10.1186/1471-230X-8-40 18782455 PMC2547101

[B9] de Lima VM Oliveira CP Alves VA Chammas MC Oliveira EP Stefano JT de Mello ES Cerri GG Carrilho FJ Caldwell SH A rodent model of NASH with cirrhosis, oval cell proliferation and hepatocellular carcinoma J Hepatol 2008 49 1055 1061 10.1016/j.jhep.2008.07.024 18929425

[B10] Dias JV Paredes BD Mesquita LF Carvalho AB Kozlowski EO Lessa AS Takiya CM Resende CM Coelho HS Campos-de-Carvalho AC Rezende GF Goldenberg RC An ultrasound and histomorphological analysis of experimental liver cirrhosis in rats Braz J Med Biol Res 2008 41 992 999 10.1590/S0100-879X2008001100008 19099152

[B11] Feeney DA Anderson KL Ziegler LE Jessen CR Daubs BM Hardy RM Statistical relevance of ultrasonographic criteria in the assessment of diffuse liver disease in dogs and cats Am J Vet Res 2008 69 212 221 10.2460/ajvr.69.2.212 18241018

[B12] Nyland TG Fisher PE Evaluation of experimentally induced canine hepatic cirrhosis using duplex Doppler ultrasound Vet Radiol 1990 31 189 194 10.1111/j.1740-8261.1990.tb01809.x

[B13] Guan S Zhao WD Zhou KR Peng WJ Mao J Tang F CT perfusion at early stage of hepatic diffuse disease World J Gastroenterol 2005 11 3465 3467 15948256 10.3748/wjg.v11.i22.3465 PMC4316005

[B14] Kreft B Dombrowski F Block W Bachmann R Pfeifer U Schild H Evaluation of different models of experimentally induced liver cirrhosis for MRI research with correlation to histopathologic findings Invest Radiol 1999 34 360 366 10.1097/00004424-199905000-00006 10226849

[B15] Kim H Booth CJ Pinus AB Chen P Lee A Qiu M Whitlock M Murphy PS Constable RT Induced hepatic fibrosis in rats: hepatic steatosis, macromolecule content, perfusion parameters, and their correlations--preliminary MR imaging in rats Radiology 2008 247 696 705 10.1148/radiol.2473070605 18403622

[B16] Quintanilha LF Mannheimer EG Carvalho AB Paredes BD Dias JV Almeida AS Gutfilen B Barbosa da Fonseca LM Resende CM Rezende GF Campos de Carvalho AC Goldenberg RC Bone marrow cell transplant does not prevent or reverse murine liver cirrhosis Cell Transplant 2008 17 943 953 10.3727/096368908786576453 19069636

[B17] Ju S Teng GJ Lu H Zhang Y Zhang A Chen F Ni Y In vivo MR tracking of mesenchymal stem cells in rat liver after intrasplenic transplantation Radiology 2007 245 206 215 10.1148/radiol.2443061290 17717324

[B18] Bedogni G Bellentani S Miglioli L Masutti F Passalacqua M Castiglione A Tiribelli C The Fatty Liver Index: a simple and accurate predictor of hepatic steatosis in the general population BMC Gastroenterol 2006 6 33 10.1186/1471-230X-6-33 17081293 PMC1636651

[B19] Hepburn MJ Vos JA Fillman EP Lawitz EJ The accuracy of the report of hepatic steatosis on ultrasonography in patients infected with hepatitis C in a clinical setting: a retrospective observational study BMC Gastroenterol 2005 5 14 10.1186/1471-230X-5-14 15829009 PMC1087838

[B20] Hong WD Zhu QH Huang ZM Chen XR Jiang ZC Xu SH Jin K Predictors of esophageal varices in patients with HBV-related cirrhosis: a retrospective study BMC Gastroenterol 2009 9 11 10.1186/1471-230X-9-11 19196464 PMC2661092

[B21] Partington BP Biller DS Hepatic imaging with radiology and ultrasound Vet Clin North Am Small Anim Pract 1995 25 305 335 7785166 10.1016/s0195-5616(95)50029-4

[B22] Mwanza T Miyamoto T Okumura M Kadosawa T Fujinaga T Ultrasonography, biochemical and hematological profiles in liver disease caused by intravenous administration of dimethylnitrosamine in dogs Jpn J Vet Res 1997 45 153 161 9433016

[B23] Biller DS Kantrowitz B Miyabayashi T Ultrasonography of diffuse liver disease. A review J Vet Intern Med 1992 6 71 76 1588544 10.1111/j.1939-1676.1992.tb03154.x

[B24] Stowater CR Lamb CH Schelling SH Ultrasonographic features of canine nodular hyperplasia Vet Radiol 1990 32 268 271 10.1111/j.1740-8261.1990.tb00800.x

[B25] Giorgio A Amoroso P Lettieri G Fico P de Stefano G Finelli L Scala V Tarantino L Pierri P Pesce G Cirrhosis: value of caudate to right lobe ratio in diagnosis with US Radiology 1986 161 443 445 3532188 10.1148/radiology.161.2.3532188

[B26] Lafortune M Constantin A Breton G Legare AG Lavoie P The recanalized umbilical vein in portal hypertension: a myth AJR Am J Roentgenol 1985 144 549 553 3881894 10.2214/ajr.144.3.549

[B27] Liao AH Cheng YC Weng CH Tsai TF Lin WH Yeh SH Yeh WC Li PC Characterization of malignant focal liver lesions with contrast-enhanced 40 MHz ultrasound imaging in hepatitis B virus X transgenic mice: a feasibility study Ultrason Imaging 2008 30 203 216 19507674 10.1177/016173460803000402

[B28] Hanajiri K Mitsui H Maruyama T Hashimoto N Sata M Omata M Echographic detection of diethylnitrosamine-induced liver tumors in rats and the effect of the intratumoral injection of an inhibitor of c-Jun N-terminal kinase J Gastroenterol Hepatol 2009 24 866 871 10.1111/j.1440-1746.2008.05722.x 19220657

[B29] Lee GP Jeong WI Jeong DH Do SH Kim TH Jeong KS Diagnostic evaluation of carbon tetrachloride-induced rat hepatic cirrhosis model Anticancer Res 2005 25 1029 1038 15868943

[B30] Matsuhashi T Yamada N Shinzawa H Takahashi T An evaluation of hepatic ultrasound speed in injury models in rats: correlation with tissue constituents J Ultrasound Med 1996 15 563 570 8839403 10.7863/jum.1996.15.8.563

[B31] Layer G Zuna I Lorenz A Zerban H Haberkorn U Bannasch P Van Kaick G Räth U Computerized ultrasound B-scan texture analysis of experimental diffuse parenchymal liver disease: correlation with histopathology and tissue composition J Clin Ultrasound 1991 19 193 201 10.1002/jcu.1870190402 1646222

[B32] Carvalho AB Quintanilha LF Dias JV Paredes BD Mannheimer EG Carvalho FG Asensi KD Gutfilen B Fonseca LM Resende CM Rezende GF Takiya CM de Carvalho AC Goldenberg RC Bone marrow multipotent mesenchymal stromal cells do not reduce fibrosis or improve function in a rat model of severe chronic liver injury Stem Cells 2008 26 1307 1314 10.1634/stemcells.2007-0941 18308943

[B33] Reeves PG Nielsen FH Fahey GC Jr AIN-93 purified diets for laboratory rodents: final report of the American Institute of Nutrition ad hoc writing committee on the reformulation of the AIN-76A rodent diet J Nutr 1993 123 1939 1951 8229312 10.1093/jn/123.11.1939

[B34] Lacaz CS Porto E Martins JEC Heins-Vaccari EM Melo NT Tratado de Micologia 1998 São Paulo: Sarvier

[B35] Dolber PC Spach MS Conventional and confocal fluorescence microscopy of collagen fibers in the heart J Histochem Cytochem 1993 41 465 469 7679127 10.1177/41.3.7679127

[B36] Ishak K Baptista A Bianchi L Callea F De Groote J Gudat F Denk H Desmet V Korb G MacSween RN Phillips MJ Portmann BG Poulsen H Scheuer PJ Schmid M Thaler H Histological grading and staging of chronic hepatitis J Hepatol 1995 22 696 699 10.1016/0168-8278(95)80226-6 7560864

[B37] Plummer JL Hall PD Ilsley AH Cmielewski PL Ahern MJ Williams RA Dose-response relationships in hepatic injury produced by alcohol and carbon tetrachloride Alcohol Clin Exp Res 1994 18 1523 1526 10.1111/j.1530-0277.1994.tb01460.x 7695054

[B38] Fletcher RH Fletcher SW Wagner EH Clinical Epidemiology: The Essentials 2005 Philadelphia: Lippincott Williams & Wilkins

[B39] Henson FM Lamas L Knezevic S Jeffcott LB Ultrasonographic evaluation of the supraspinous ligament in a series of ridden and unridden horses and horses with unrelated back pathology BMC Vet Res 2007 3 3 10.1186/1746-6148-3-3 17331234 PMC1821016

[B40] Tchelepi H Ralls PW Radin R Grant E Sonography of diffuse liver disease J Ultrasound Med 2002 21 1023 1032 12216750 10.7863/jum.2002.21.9.1023

[B41] Lamb CR Abdominal ultrasonography in small animals: examination of the liver, spleen and pancreas J Small Anim Pract 1990 31 5 8 10.1111/j.1748-5827.1990.tb00645.x

[B42] Schalm SW The diagnosis of cirrhosis: clinical relevance and methodology J Hepatol 1997 27 1118 1119 10.1016/S0168-8278(97)80159-6 9453441

[B43] Hall PD Plummer JL Iisley AH Cousins MJ Hepatic fibrosis and cirrhosis after chronic administration of alcohol and "low-dose" carbon tetrachloride vapor in the rat Hepatology 1991 13 815 819 10.1016/0270-9139(91)90246-R 2029987

[B44] Plummer JL Hall PD Cmielewski PL Iisley AH Ahern MJ Alcohol/"Low-dose" carbon tetrachloride-induced cirrhosis in rats using different methods of alcohol feeding Acohol Clin Exp Res 1994 18 1502 1505 10.1111/j.1530-0277.1994.tb01457.x 7695051

[B45] Siegers CP Völpel M Scheel G Younes M Effects of dithiocarb and (+)-catechin against carbon tetrachloride-alcohol-induced liver fibrosis Agents Actions 1982 12 743 748 10.1007/BF01965096 6299080

[B46] Muñoz Torres E Paz Bouza JI Abad Hernandez MM Alonso Martin MJ Lopez Bravo A Experimental carbon tetrachloride-induced cirrhosis of the liver Int J Tissue React 1988 10 245 251 3250938

[B47] Dick R Sutton D The liver and spleen Textbook of radiology imaging 1998 6 China: Churchill Livingstone 981 1028

[B48] England GCW Renal and hepatic ultrasonography in the neonatal dog Vet Radiol Ultrasound 1996 37 374 382 10.1111/j.1740-8261.1996.tb01246.x

[B49] Ivancic M Mai W Qualitative and quantitative comparison of renal vs. hepatic ultrasonographic intensity in healthy dogs Vet Radiol Ultrasound 2008 49 368 373 10.1111/j.1740-8261.2008.00383.x 18720770

[B50] Drost WT Henry GA Meinkoth JH Woods JP Lehenbauer TW Quantification of hepatic and renal cortical echogenicity in clinically normal cats Am J Vet Res 2000 61 1016 1020 10.2460/ajvr.2000.61.1016 10976729

[B51] Mathiesen UL Franzen LE Aselius H Resjo M Jacobsson L Foberg U Fryden A Bodemar G Increased liver echogenicity at ultrasound examination reflects degree of steatosis but not of fibrosis in asymptomatic patients with mild/moderate abnormalities of liver transaminases Dig Liver Dis 2002 34 516 522 10.1016/S1590-8658(02)80111-6 12236486

[B52] Palmentieri B de Sio I La Mura V Masarone M Vecchione R Bruno S Torella R Persico M The role of bright liver echo pattern on ultrasound B-mode examination in the diagnosis of liver steatosis Dig Liver Dis 2006 38 485 489 10.1016/j.dld.2006.03.021 16716779

[B53] Colli A Fraquelli M Andreoletti M Marino B Zuccoli E Conte D Severe liver fibrosis or cirrhosis: accuracy of US for detection--analysis of 300 cases Radiology 2003 227 89 94 10.1148/radiol.2272020193 12601199

[B54] Voros K Albert M Vetesi F Harmat G Binder K Szaniszlo F Hepatic ultrasonographic findings in experimental carbon tetrachloride intoxication of the dog Acta Vet Hung 1997 45 137 150 9270137

